# Sterilization of Tuberculous Meat

**Published:** 1896-04

**Authors:** 


Sterilization of Tuberculous Meat. According to ministerial decision of September 30, 1895, it has been enacted in Belgium that meat coming from tuberculous cattle, which otherwise would have to be discarded, can be sold for consumption in case it has been exposed to a moist temperature of 110° C. under the supervision of a veterinary surgeon for at least three hours in a sterilizer which is approved by the Minister of Agriculture and Public Works.—*Tijdschrift voor Veeartsenijkunde en Veeteelt*, January, 1896.

